# Persistent Right Umbilical Vein in Association With Single Umbilical Artery: A Case Report and Review of Literature

**DOI:** 10.7759/cureus.36544

**Published:** 2023-03-22

**Authors:** Ipsita Mohapatra, Subha R Samantaray

**Affiliations:** 1 Obstetrics and Gynecology, All India Institute of Medical Sciences Kalyani, Kalyani, IND

**Keywords:** single umbilical artery, prelabour premature rupture of membranes, cardiovascular anomalies, vascular malformations, persistent right umbilical vein

## Abstract

Persistent right umbilical vein (PRUV) and single umbilical artery (SUA) are developmental malformations of the vascular system. In isolation, these are not rare, but the presence of these two malformations together is not very common. When they are present together, there are increased chances of associated congenital anomalies, especially anomalies of the vascular system. So, when these two coexist, a detailed examination of all other organ systems, especially the cardiovascular system, should be conducted. The accurate evaluation of such vascular malformations during fetal life is needed to provide adequate antenatal counseling, the timing of delivery, and appropriate post-natal care. We report a case of a primigravida who was diagnosed with PRUV and SUA in the fifth month of gestation. In this article, we discuss this case's management with a literature review. The anomaly scan done at around 21 weeks revealed a two-vesseled umbilical cord with an SUA and PRUV. Apart from this, there were no other structural anomalies. The patient had preterm delivery at 35 weeks 5 days gestation period and delivered a 2.6 kg male baby.

## Introduction

Persistent right umbilical vein (PRUV) is a developmental malformation of the vascular system during the embryonic period where in the left umbilical vein becomes atretic and the right umbilical vein remains open [1]. During normal development, the right umbilical vein slowly becomes atretic at the embryonic fourth week and completely disappears by the seventh week. There is degeneration of the segment of the left umbilical vein between the liver and the sinus venosus. Only the portion of the left umbilical vein which is remaining between the umbilicus and the liver persists and communicates with the umbilical vein in the umbilical cord [2].

PRUV is an uncommon condition, and its reported incidence varies between one in 250 to one in 1,250 [3]. PRUV has two types of presentations. The intrahepatic PRUV is the most common (90%-95%) while the extrahepatic type is less common, but most frequently associated with complications [4].

The other frequent vascular malformation of the umbilical cord is the single umbilical artery (SUA), a condition where a single umbilical artery is present in the umbilical cord instead of two. It is found in about 0.5%-6% of pregnancies [5,6].

About 65% of cases with SUA and 74.8% of cases with PRUV are reported to be isolated findings without any type of associated malformations [5]. SUA has been associated with an increase in the risk of other malformations especially cardiovascular anomalies in PRUV fetuses [7]. Both these conditions have been associated with intrauterine growth restriction, oligohydramnios, and preterm deliveries. In this report, we are presenting a case of 28 years primigravida, who presented with a single live fetus with PRUV and SUA at the fifth month of gestation and its management.

## Case presentation

**Figure 1:**
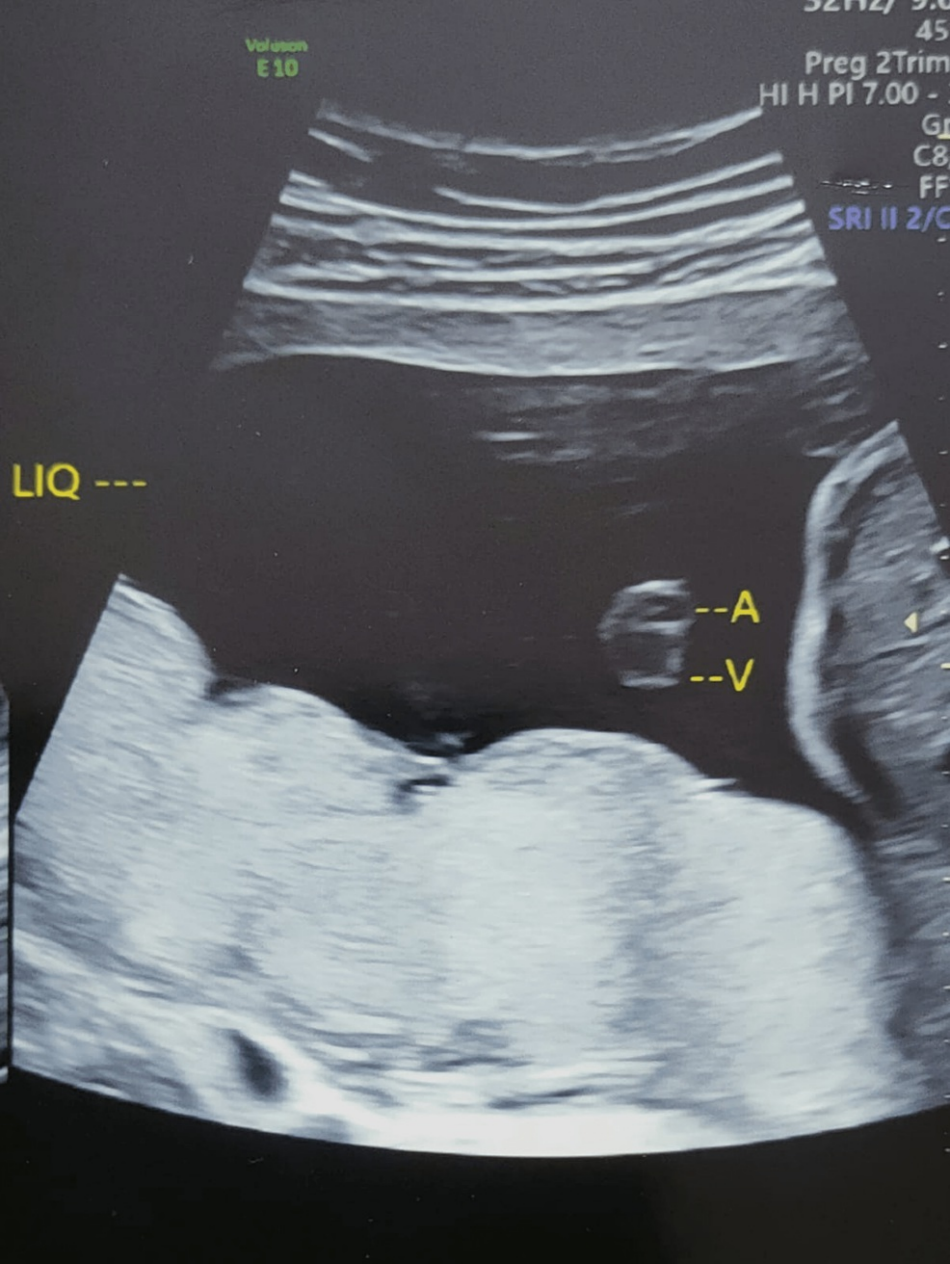
Cross-section of umbilical cord showing two vessels

**Figure 2:**
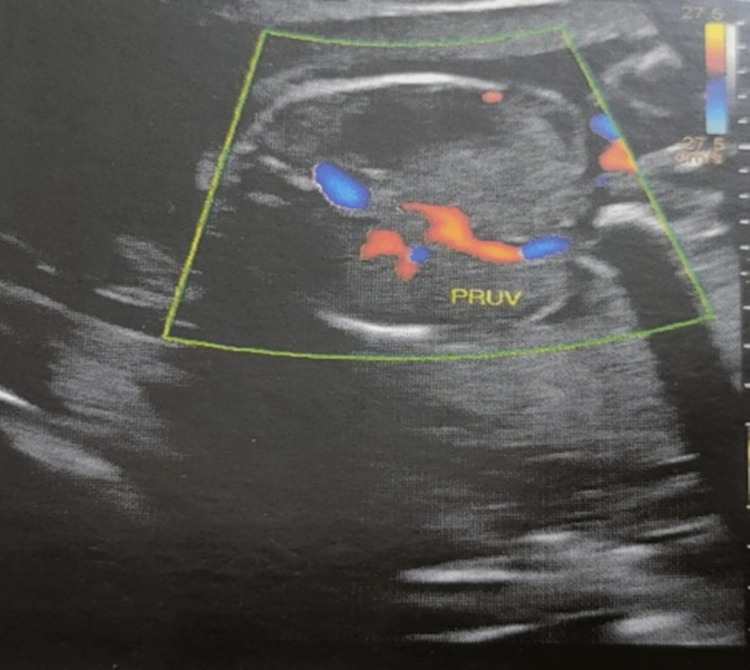
The umbilical vein bending toward the stomach in the abdominal circumference section

A 28 years primigravida came to our department for the first antenatal checkup at six weeks of gestational age. Routine investigations during the first trimester were normal. The patient was advised nuchal translucency scan and double marker test in the late first trimester. Nuchal translucency was 1.69mm and ductus venosus flow pattern was within normal limits. An Anomaly scan was done at around 21 weeks of gestation which revealed two-vesseled umbilical cord (Figure 1) on cross-section with a SUA and persistent right umbilical vein (PRUV) (Figure 2). There was no other structural abnormality detected.

The condition was discussed with the couple and the possibility of some associated anomalies, development of intrauterine growth restriction (IUGR), or preterm labor was discussed. Chromosomal microarray analysis was done which did not reveal any copy number variations of clinical significance. Fluorescence in situ hybridization (FISH) was performed on interphase nuclei from an uncultured amniotic fluid sample of this patient using LSI 13/18/ 21 and CEP sex chromosome probes from Metasystems, Germany, localized to 13q14, 18q21, 21q22 and centromeric region of sex chromosomes. No aneuploidy was detected for 13, 18, 21, and Sex chromosomes.

Fetal two-dimensional echocardiography was done at 28 weeks of gestation to rule out any associated cardiac anomalies which are the most common type of anomalies in cases with PRUV and SUA. The report was normal and no cardiac anomalies were detected.

The patient was followed up with more frequent antenatal visits keeping in mind the possibility of the development of IUGR and oligohydramnios. The fetus's development and growth were within normal limits and the amniotic fluid volume remained normal. But at 35 weeks and 5 days gestational period, the patient presented with prelabor premature rupture of membranes (PPROM). The cause of PPROM could not be elicited. After corticosteroid prophylaxis for fetal lung maturity, the patient was delivered by cesarean section due to nonprogress of labor. She delivered a 2.6 kg male baby. The baby developed physiological jaundice on the fourth postnatal day and was managed conservatively. The baby is now three months old and doing fine. The patient was informed, and consent was taken before submitting this case report.

## Discussion

The formation of the umbilical cord occurs between day 13 to day 38 post-conception and it consists of three vessels, one vein, and two arteries. But in the case of primary agenesis, secondary atresia, or persistent allantoic artery of the body stalk, there may presence of some vascular malformations like SUA or PRUV [3]. SUA and PRUV are the most common types of developmental vascular malformations of the umbilical cord.

PRUV has two types-the intrahepatic variant and the extrahepatic variant. The intrahepatic PRUV is more common. In the intrahepatic variant (I-PRUV), there is a fusion of the right umbilical vein with the right portal vein and then it drains into the inferior vena cava through the ductus venous. It has a good prognosis and is less commonly associated with other malformations. The extrahepatic variant is less common. In this, the right umbilical vein completely bypasses the liver and directly drains into the right atrium or the intracardiac portion of the inferior vena cava or the iliac veins [3]. It is more commonly associated with other anomalies and fetal congestive heart failure due to cardiac overload and hemodynamic stresses.

The incidence of PRUV has increased in recent years due to better ultrasonographic diagnosis. Most of the cases of PRUV are not associated with any other anomaly. Common malformations which may be associated with PRUV are cardiac, nervous, skeletal, and genitourinary. The malformation rate is about 13%-40.9% [7]. Among the associated cardiac abnormalities, the ventricular septal defect has been found to be the most common [8].

SUA is a common vascular malformation with an incidence of about 0.5%-6% worldwide [9]. In about 30%-60% of SUA cases, some congenital abnormality may be associated with cardiac, genitourinary, skeletal, gastrointestinal, or chromosomal abnormalities like trisomy 13, 18, 21, and triploidy [10]. Some syndromes like VATER complex (vertebral defects, imperforate anus, tracheoesophageal fistula, and radial and renal dysplasia) and Meckel-Gruber and Zellweger syndrome have been associated with SUA [10].

The etiology for the development of vascular developmental malformations is not established. Some reports suggest that thromboembolism or external compression of any cause in early pregnancy may result in blockage of the left umbilical vein leading to the continuation of blood supply in the right umbilical vein [2]. Some other reports suggest that inadequate supplementation of folic acid or use of any specific teratogenic drug in the early trimester may result in PRUV [11].

The diagnosis of PRUV can be done during prenatal ultrasonography by examining the transverse section of the fetal abdomen if any of the three criteria are met: (1) the portal vein is found to curve towards the stomach, or (2) the gall bladder of the fetus is found medial to the umbilical vein or (3) the umbilical vein is connected abnormally to the right portal vein [12]. A PRUV is oriented towards the stomach and not toward the right lobe of the liver in the plane in which the hockey stick appearance is seen. After the diagnosis of PRUV is made, it is important to look for the location of the ductus venosus so that PRUV can be differentiated into intra or extrahepatic variants. Fetal echocardiography is also recommended to rule out any other associated anomalies.

SUA is diagnosed in the first or second trimester, at the level of the fetal bladder when an umbilical artery is found on only one side of the bladder. In the third trimester, the normal umbilical cord shows the Mickey Mouse sign, but in the case of SUA, one ear of the Mickey Mouse is found to be missing.

According to one study, in PRUV cases; SUA was associated with a higher incidence of cardiovascular anomalies [7]. Our case had the presence of PRUV associated with SUA. The fetal echocardiography, FISH, and chromosomal microarray analysis did not reveal any associated structural or chromosomal anomaly. In cases with PRUV and SUA careful fetal monitoring and care are needed, especially in extrahepatic PRUV to prevent congestive heart failure and hydrops [13].

Cesarean section is not indicated for isolated cases of SUA in order to avoid potential fetal distress during vaginal delivery. The present case had PPROM at 35 weeks and was delivered by cesarean section due to non-progress of labor. While some articles have concluded that rate of preterm delivery and fetal distress doesn’t increase in cases of isolated cases PRUV and SUA, some others suggest that these complications may be seen more frequently [12,14].

## Conclusions

The presence of SUA along with PRUV is not common. When these two coexist, a detailed examination of other organ systems, especially the cardiovascular system, should be conducted. The accurate evaluation of such vascular malformations during fetal life is needed to provide adequate antenatal counseling, the timing of delivery, and appropriate post-natal care.
